# Supplementary material for: DNA Methylation in Neurodegenerative and Cerebrovascular Disorders
Source: Int J Mol Sci. 2020 Mar 23;21(6):2220. doi: 10.3390/ijms21062220 (PMC7139499; doi:10.3390/ijms21062220)
Supplement: Supplementary file 1 [file ijms-21-02220-s001.pdf]

Supplementary Materials:

DNA methylation in Neurodegenerative and Cerebrovascular Diseases Disorders

Olaia Martínez-Iglesias *, Iván Carrera, Juan Carlos Carril, Lucía Fernández-Novoa, Natalia Cacabelos and Ramón Cacabelos

SUPPLEMENTARY MATERIALS AND METHODS

Histograms. Histograms with all data in the different pathologies were performed with GraphPad Prisma software.

Regression analysis. Analysis of correlation between 5mC or 5hmC levels and parameters like age, sex, psychometric parameters and APOE genotype was performed with regression studies in Microsoft Excell software. R2 value is indicated in graphics. P values are also indicated in graphics when this value is significant or closely to be significant. *p* value is indicated in the figures as * $p< 0.05$, **$p < 0.01$ and *** $p < 0.001$.

SUPPLEMENTARY FIGURE LEGENDS

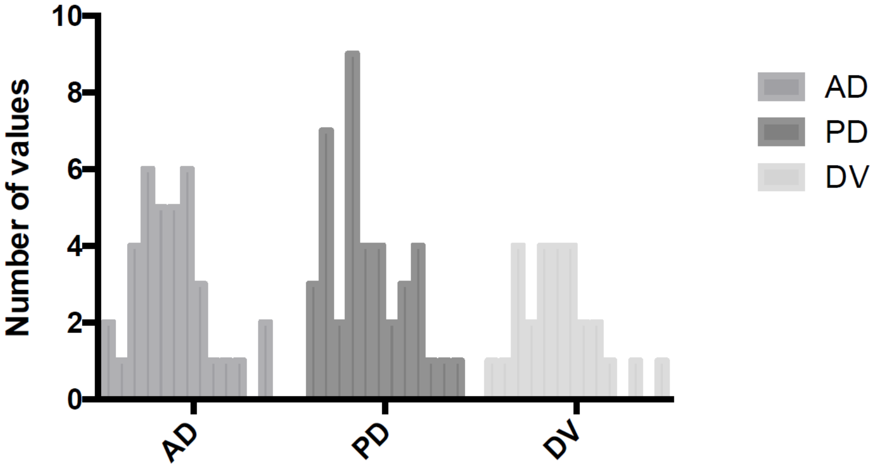

Figure S1. Histograms of 5mC values obtained in AD, PD, and DV samples. All values obtained were represented in a histogram with GraphPad Prisma software.

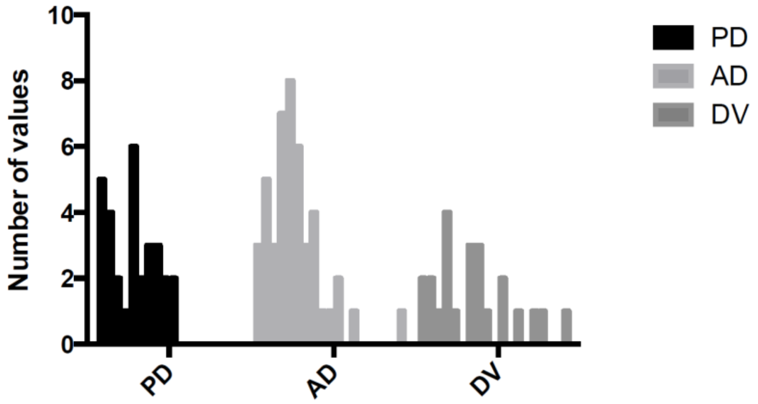

Figure S2. Histograms of 5hmC values obtained in AD, PD, and DV samples. All values obtained were represented in a histogram with GraphPad Prisma software.

A)

		Healthy	AD	PD	DV
22	Shapiro-Wilk normality test				
23	W	0.9594	0.9491	0.9492	0.9656
24	P value	0.1500	0.0900	0.0605	0.4912
25	Passed normality test (alpha=0.05)?	Yes	Yes	Yes	Yes
26	P value summary	ns	ns	ns	ns

B)

		Healthy	AD	PD	DV
22	Shapiro-Wilk normality test				
23	W	0.9386	0.9243	0.9509	0.9310
24	P value	0.0562	0.0935	0.0882	0.1149
25	Passed normality test (alpha=0.05)?	Yes	Yes	Yes	Yes
26	P value summary	ns	ns	ns	ns

C)

		Healthy	PD	Dementia
22	Shapiro-Wilk normality test			
23	W	0.9245	0.8926	0.8432
24	P value	0.5055	0.2472	0.1063
25	Passed normality test (alpha=0.05)?	Yes	Yes	Yes
26	P value summary	ns	ns	ns

Figure S3. Normality test results. Shapiro-Wilk test was used to check normal distribution with Prisma GraphPad software in global DNA methylation (**A**), hydroxymethylation (**B**) and DNMT3a expression (**C**) studies.

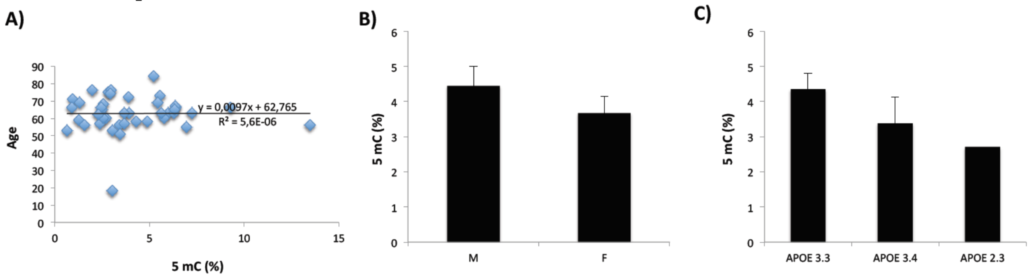

Figure S4. Correlation studies between 5mC levels and sample characteristics in Healthy samples. (**A**) Regression studies between 5mC levels and age. (**B**) Comparation of 5mC levels between males and females. (**C**) Comparation of 5mC levels between the different APOE genotypes.

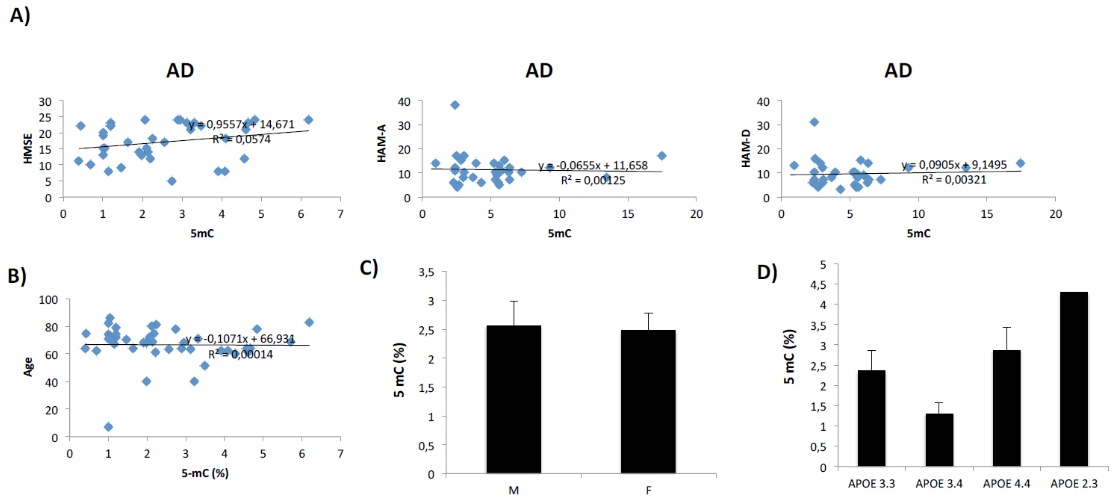

Figure S5. Correlation studies between 5mC levels and sample characteristics in AD group. (**A**) Regression studies between 5mC levels and different psychometric parameters. (**B**) Similar to (A) between 5mC levels and age. (**C**) Comparation of 5mC levels between males and females. (**D**) Comparation of 5mC levels between the different APOE genotypes.

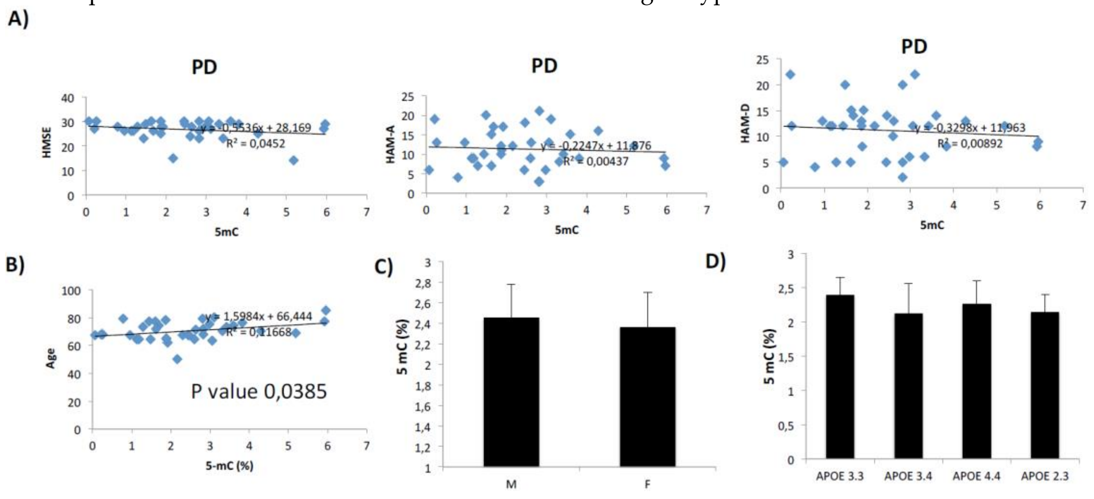

Figure S6. Correlation studies between 5mC levels and sample characteristics in PD group. (**A**) Regression studies between 5mC levels and different psychometric parameters. (**B**) Similar to (A) between 5mC levels and age. (**C**) Comparation of 5mC levels between males and females. (**D**) Comparation of 5mC levels between the different APOE genotypes. P values are also indicated in graphics when this value is significant or closely to be significant. p value is indicated in the figures as * $p < 0.05$, ** $p < 0.01$ and *** $p < 0.001$.

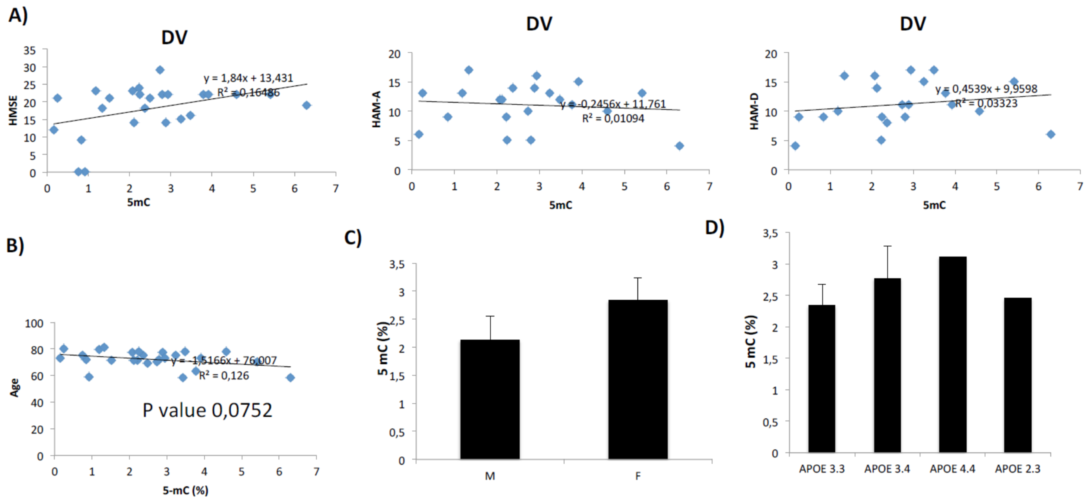

Figure S7. Correlation studies between 5mC levels and sample characteristics in DV group. (**A**) Regression studies between 5mC levels and different psychometric parameters. (**B**) Similar to (A) between 5mC levels and age. (**C**) Comparation of 5mC levels between males and females. (**D**) Comparation of 5mC levels between the different APOE genotypes. P values are also indicated in graphics when this value is significant or closely to be significant. p value is indicated in the figures as * $p < 0.05$, ** $p < 0.01$ and *** $p < 0.001$.

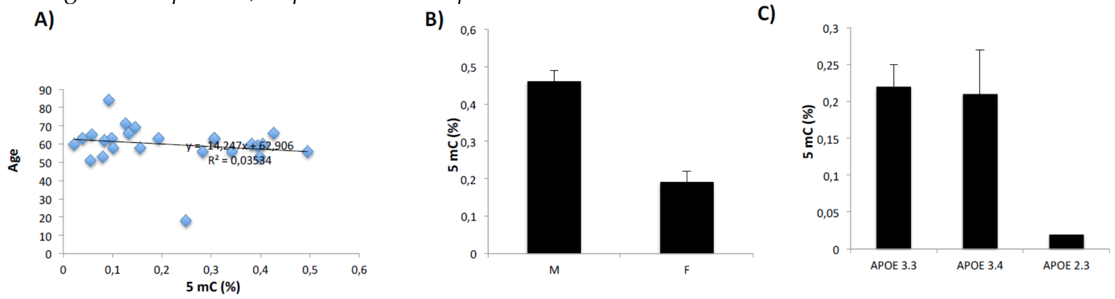

Figure S8. Correlation studies between 5hmC levels and sample characteristics in Healthy subjects. (**A**) Regression studies between 5hmC levels and age. (**B**) Comparation of 5hmC levels between males and females. (**C**) Comparation of 5hmC levels between the different APOE genotypes.

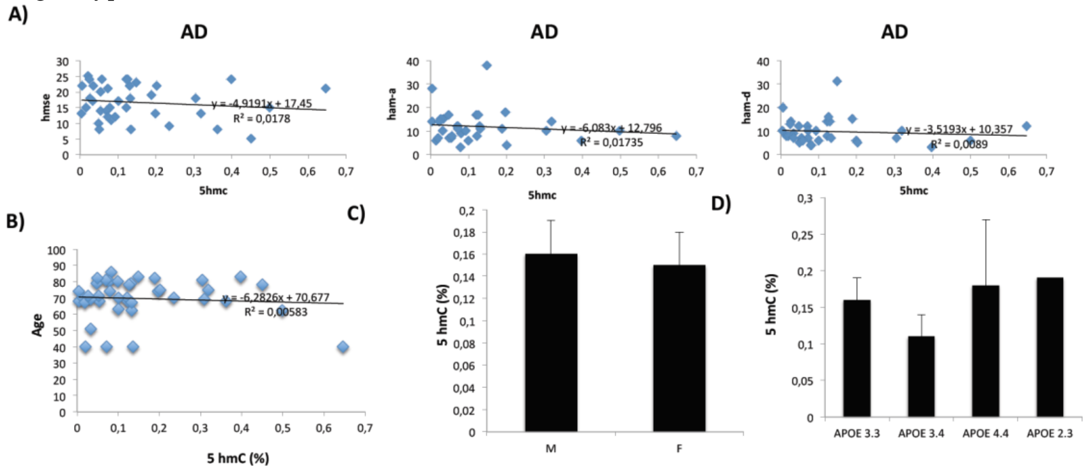

Figure S9. Correlation studies between 5hmC levels and sample characteristics in AD group. (**A**) Regression studies between 5hmC levels and different psychometric parameters. (**B**) Similar to (A) between 5hmC levels and age. (**C**) Comparation of levels between males and females. (**D**) Comparation of 5hmC levels between the different APOE genotypes. *p* values are also indicated

in graphics when this value is significant or closely to be significant. p value is indicated in the figures as * $p < 0.05$, ** $p < 0.01$ and *** $p < 0.001$.

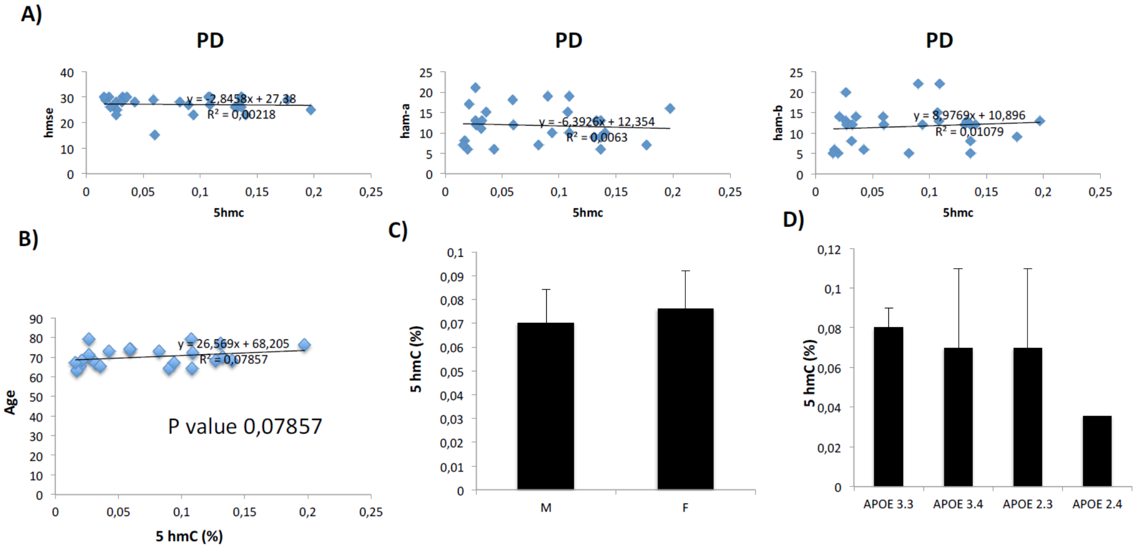

Figure S10. Correlation studies between 5hmC levels and sample characteristics in PD group. (**A**) Regression studies between 5mC levels and different psychometric parameters. (**B**) Similar to (A) between 5mC levels and age. (**C**) Comparation of 5mC levels between males and females. (**D**) Comparation of 5mC levels between the different APOE genotypes. *p* values are also indicated in graphics when this value is significant or closely to be significant. *p* value is indicated in the figures as * $p < 0.05$, ** $p < 0.01$ and *** $p < 0.001$.

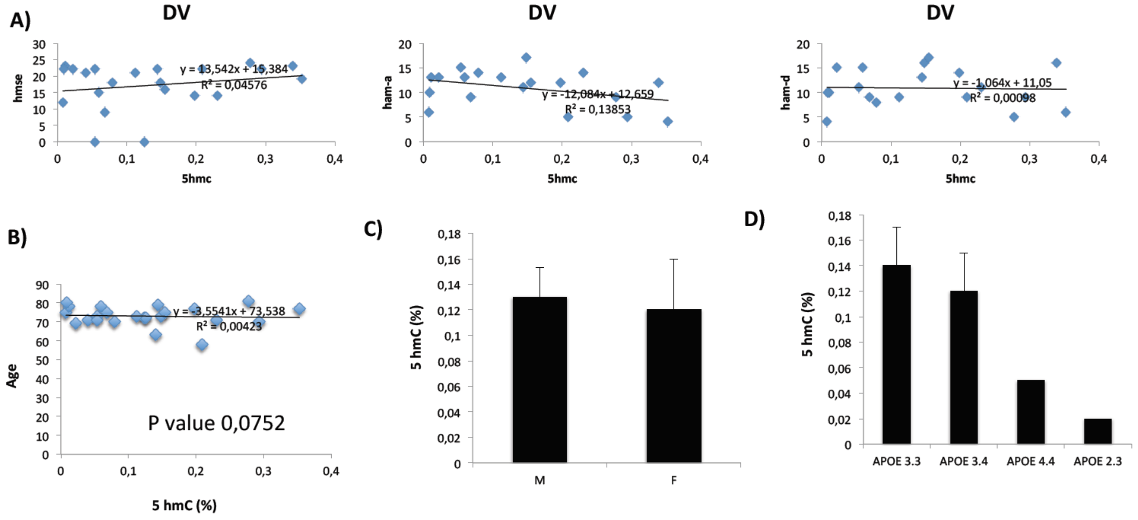

Figure S11. Correlation studies between 5hmC levels and sample characteristics in DV group. (**A**) Regression studies between 5mC levels and different psychometric parameters. (**B**) Similar to (A) between 5mC levels and age. (**C**) Comparation of 5mC levels between males and females. (**D**) Comparation of 5mC levels between the different APOE genotypes. *p* values are also indicated in graphics when this value is significant or closely to be significant. *p* value is indicated in the figures as * $p < 0.05$, ** $p < 0.01$ and *** $p < 0.001$.

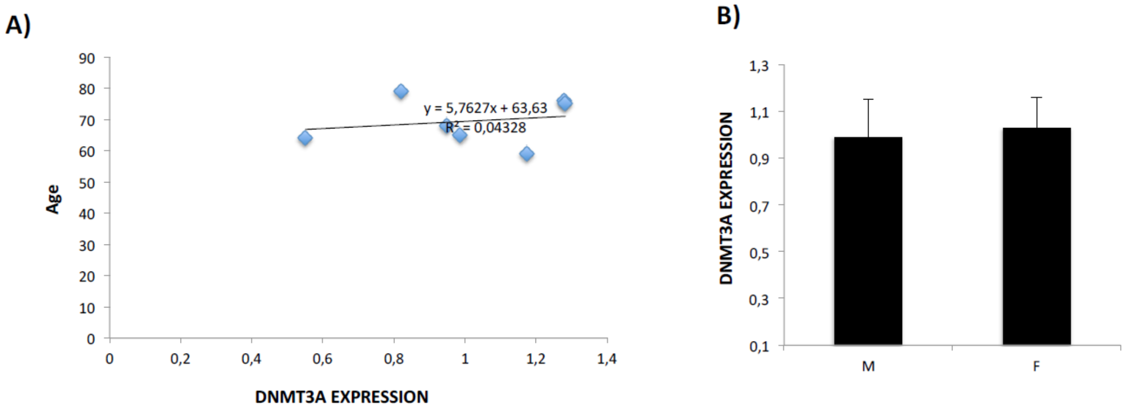

Figure S12. Correlation studies between DNMT3a expression and sample characteristics in Healthy samples. (**A**) Regression studies between DNMT3a expression and age. (**B**) Comparation of 5mC levels between males and females.

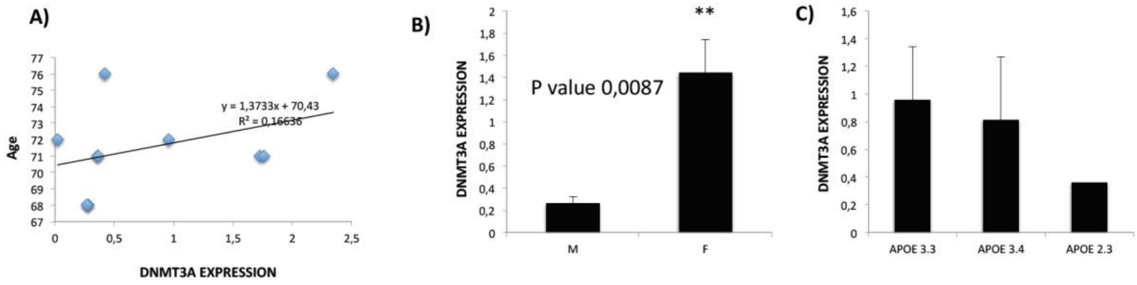

Figure S13. Correlation studies between DNMT3a expression and sample characteristics in PD. (**A**) Regression studies between DNMT3a expression and age. (**B**) Comparation of 5mC levels between males and females. (**C**) Comparation of 5mC levels in the different APOE genotypes. *p* values are also indicated in graphics when this value is significant or closely to be significant. *p* value is indicated in the figures as * $p < 0.05$, ** $p < 0.01$ and *** $p < 0.001$.

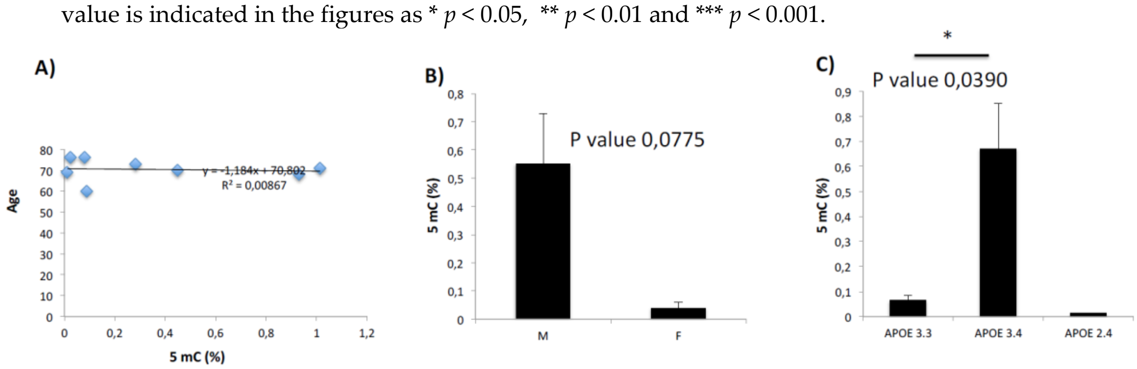

Figure S14. Correlation studies between DNMT3a expression and sample characteristics in Dementia´s group. (**A**) Regression studies between DNMT3a expression and age. (**B**) Comparation of 5mC levels between males and females. (**C**) Comparation of 5mC levels in the different APOE genotypes. *p* values are also indicated in graphics when this value is significant or closely to be significant. *p* value is indicated in the figures as * $p < 0.05$, ** $p < 0.01$ and *** $p < 0.001$.
